# Zooming in on Agoraphobic Behaviors: a Case Study

**DOI:** 10.1192/j.eurpsy.2022.1468

**Published:** 2022-09-01

**Authors:** C. Solis, R. De Sousa, T. Marques

**Affiliations:** ACeS Baixo Mondego, Usf Coimbra Centro, Coimbra, Portugal

**Keywords:** Depression, agoraphobia, e-medicine, pandemic

## Abstract

**Introduction:**

The COVID-19 pandemic brought many new challenges that people had to overcome with ingenuity. However, many patients already suffering with psychiatric diseases saw their access to conventional health care limited, aggravating their statuses. E-Medicine is the branch of health care that provides access through the Internet, and it has been growing in the last few years. During the COVID-19 pandemic, many health care workers shifted towards E-Medicine, aiming to provide support to patients, especially with the social distance policies that were implemented worldwide.

**Objectives:**

Provide an example of how e-Medicine can be a tool in establishing a therapeutic alliance, and patient follow-up

**Methods:**

Case report with a brief literature review on the subject

**Results:**

RG is a 19-year-old female that contacts her family doctor through e-mail, expressing concerns over not being able to leave her house for over a year, also manifesting anxiety and depressed humor. This started in April 2020 and was slowly worsening throughout the year, culminating in a panic attack. RG started counselling and follow-up appointments via Internet and started treatment with vortioxetine. Three months later, improvements were stated, namely decreased anxiety, better sleep patterns, and leaving the house for small periods.

**Conclusions:**

Without E-Medicine, RG wouldn’t be as able to reach out to her family doctor, and follow-up would be much more arduous since the patient avoided leaving her house, and telephone appointments lack the visual aspect of the clinical interview. E-Medicine is a valid alternative to conventional Medicine, providing a safe environment for patients concerned with public space.

**Disclosure:**

No significant relationships.

